# Challenges and Advancement of Blue III-Nitride Vertical-Cavity Surface-Emitting Lasers

**DOI:** 10.3390/mi12060676

**Published:** 2021-06-09

**Authors:** Chia-Yen Huang, Kuo-Bin Hong, Zhen-Ting Huang, Wen-Hsuan Hsieh, Wei-Hao Huang, Tien-Chang Lu

**Affiliations:** Department of Photonics, College of Electrical and Computer Engineering, National Yang Ming Chiao Tung University, Hsinchu 30010, Taiwan; kbhong@nctu.edu.tw (K.-B.H.); psgchsfmax8@gmail.com (Z.-T.H.); clock1597531@gmail.com (W.-H.H.); winjr10595@gmail.com (W.-H.H.)

**Keywords:** GaN, III-nitrides, VCSEL, distributed Bragg reflector, carrier aperture, polarization

## Abstract

Since the first demonstration of (Al, In, Ga)N-based blue vertical-cavity surface-emitting lasers (VCSELs) in 2008, the maximum output power (P_max_) and threshold current density (J_th_) has been improved significantly after a decade of technology advancements. This article reviewed the key challenges for the realization of VCSELs with III-nitride materials, such as inherent polarization effects, difficulties in distributed Bragg’s reflectors (DBR) fabrication for a resonant cavity, and the anti-guiding effect due to the deposited dielectrics current aperture. The significant tensile strain between AlN and GaN hampered the intuitive cavity design with two epitaxial DBRs from arsenide-based VCSELs. Therefore, many alternative cavity structures and processing technologies were developed; for example, lattice-matched AlInN/GaN DBR, nano-porous DBR, or double dielectric DBRs via various overgrowth or film transfer processing strategies. The anti-guiding effect was overcome by integrating a fully planar or slightly convex DBR as one of the reflectors. Special designs to limit the emission polarization in a circular aperture were also summarized. Growing VCSELs on low-symmetry non-polar and semipolar planes discriminates the optical gain along different crystal orientations. A deliberately designed high-contrast grating could differentiate the reflectivity between the transverse-electric field and transverse-magnetic field, which restricts the lasing mode to be the one with the higher reflectivity. In the future, the III-nitride based VCSEL shall keep advancing in total power, applicable spectral region, and ultra-low threshold pumping density with the novel device structure design and processing technologies.

## 1. Introduction

(Al,Ga,In)N-based laser diodes (LD) have been used in optical storage, signage, projector display, industrial manufacturing, and many other applications. The realization of III-nitride-based LDs comes much later than other arsenide- and phosphide-based LDs although they shared similar device design concepts. InGaN/GaN-based LDs were firstly demonstrated in the early 90′s after Dr. Shuji Nakamura achieved breakthroughs in epitaxial technologies [1,2,3]. Later in 90′s, the development of low threading dislocation GaN substrates further boosted the performance of nitride-based LDs [4,5,6,7]. To date, low-threshold (J_th_ < 2 kA/cm^2^) and state-of-art high-power (P > 10 Watt) edge-emitting lasers (EEL) have been demonstrated [8,9,10]. III-nitride vertical-cavity surface-emitting lasers (VCSELs) arrived even later. Electrically pumped III-nitride VCSELs were firstly demonstrated by NCTU and Nichia Corp., Tokushima, Japan, with different device configurations in 2008, which was more than a decade later than the EELs [11,12]. The late arrival of III-nitride VCSELs can be attributed to the inherent challenges from the nitride materials’ properties. Although some challenges were also encountered by EELs, the influences are more significant for VCSELs.

**Figure 1:**
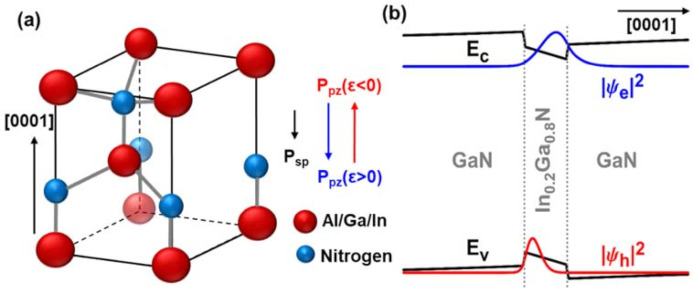
(**a**) Schematic Wurtzite nitride structure and the direction of spontaneous polarization (P_sp_) and piezoelectric polarization (P_pz_). (**b**) Band diagram of undoped 3 nm GaN/In_0.2_Ga_0.8_N/GaN quantum-well grown along c-axis with wavefunctions in a ground state.

**Figure 2:**
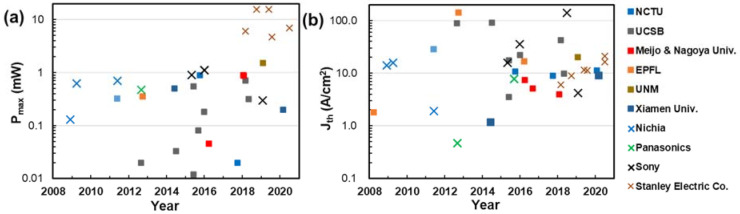
Benchmarks of (**a**) maximum output power (P_max_) and (**b**) threshold current density (J_th_) of electrically pumped nitride blue VCSELs in chronological order of publication. Device parameters were extracted from references [11,12,21,22,23,24,25,26,27,28,29,30,31,32,33,34,35,36,37,38,39,40,41,42,43,44,45,46,47,48,49,50,51,52,53].

Wurtzite III-nitrides (as shown in Figure 1a) is a piezoelectric material family with distinctive differences in fundamental material parameters. Although the spontaneous polarization (P_sp_) all points toward the N-polar surface, the final polarization was mostly dominated by its piezoelectric polarization (P_pz_):

(1) $$P_{pz}=2\left(e_{31}-e_{33}\frac{C_{13}}{C_{33}}\right)*\varepsilon$$

(2) $$\varepsilon =\frac{a_{f}-a_{0}}{a_{0}}$$

where e_31_ and e_33_ are piezoelectric tensor elements, C_13_ and C_33_ are elastic constants, ε is the strain state of materials, a_f_ and a_0_ is the lattice constant in the film and in the bulk crysl, respectively. The values of the above parameters are summarized in Table 1 [13,14,15,16,17]. Since the bracket in Equation (1) is always negative, the P_pz_ is determined by the sign of ε. For AlGaN alloys on GaN template, ε>0 (tensile strain) and P_pz_ is parallel to the P_sp_; for InGaN alloys, ε<0 (compressive strain) and p_pz_ are antiparallel to the P_sp_. The total polarization is the sum of P_sp_ and P_pz_. As a result, every layer in the epi structure possesses different polarizations. The discontinuity of polarization induces sheet charges at interfaces and a zig-zag profile in a band diagram, as illustrated in Figure 1b. The zig-zag band profile lowered the overlapping between wavefunctions in the conduction band and valance band, so the transition matrix elements in the quantum well are also reduced. The wavefunction overlapping in an undoped 3 nm In_0.2_Ga_0.8_N/GaN quantum well is only ~0.15 under equilibrium, and still <0.4 under a 1 kA/cm^−2^ current injection [18]. Fortunately, the high density-of-state of III-nitrides compensated the effects of the low transition matrix element in the maximum optical gain (g_max_). The tolerable internal and scattering losses of III-nitride VCSELs are no less than those made with arsenide- or phosphide-based materials [19,20]. With the same level of optical losses in the device, the J_th_ of nitride-based VCSELs is inevitably higher. Figure 2 benchmarks the maximum output power (P_max_) and the J_th_ performance from literature in chronological order [21,22,23,24,25,26,27,28,29,30,31,32,33,34,35,36,37,38,39,40,41,42,43,44,45,46,47,48,49,50,51,52,53]. Representative device structures such as reflector types, cavity lengths, and aperture design were summarized in Table 2. From 2008 to 2020, the P_max_ has been enhanced about 100 folds for a single VCSEL, but the J_th_ remained ~10 kA/cm^−2^. The relatively limited J_th_ improvement might be also attributed to the high density of the state of III-nitride materials and the relatively short gain path in the cavity. As references, the representative J_th_ (P_max_) was 0.9 kA/cm^2^ (9 mW) for 850 nm GaAs-based VCSELs, 0.8~1.7 kA/cm^2^ (7.7~11 mW) for 940 nm~980 nm GaAs-based VCSELs, 3.1 kA/cm^2^ (3.6 mW) for 1.3 μm GaAs-based VCSEL, and 3.5 kA/cm^2^ (3.0 mW) for 1.55 μm for InP-based VCSELs [54,55,56,57,58].

Besides the strain-induced polarizations and relevant quantum confinement Stark effects (QCSE), the large lattice strain between AlN and GaN itself hampered the realization of nitride VCSELs. Unlike the AlAs is nearly strain-free on GaAs, AlN on GaN suffers from a 2.47% tensile strain. Therefore, nitride-based VCSEL could not afford a double epitaxial distributed Bragg’s reflector (DBR) mirrors as arsenide-based VCSELs do. In fact, growing a single AlN/GaN DBR with high reflectivity is particularly challenging. The low refractive index contrast further enhances the required total thickness for a high-reflectivity DBR. Table 3 summarized the refractive indices of AlN, GaN, Al_0.82_In_0.12_N, Al_0.6_Ga_0.4_N, and other reported dielectrics for nitride VCSELs at 420 nm [59,60,61,62]. We assumed a perfect Braggs condition at λ_B_ = 420 nm under room temperature and neglect the index dispersion. The reflectance (R) and stop-band width (Δλ_stop_) can be calculated by:

(3) $$R={\left[\frac{n_{2}^{2m}-n_{1}^{2m}}{n_{2}^{2m}+n_{1}^{2m}}\right]}^{2}$$

(4) $$\Delta \lambda_{stop}=\frac{4\lambda_{B}}{\pi }arcsin\left(\frac{n_{2}-n_{1}}{n_{2}+n_{1}}\right)$$

**Figure 3:**
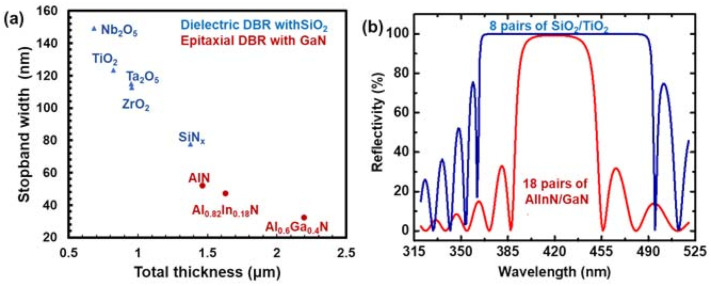
(**a**) Scatter plot of stopband width and a total thickness of ideal epitaxial and dielectric DBRs with R > 0.99 (**b**) Calculated reflectivity spectrum of selected DBRs.

*n*_2_ is the high refractive index in the DBR pair, which is usually the index of GaN in epitaxial DBRs; n_1_ is the low refractive index, which is usually the index of SiO_2_ in dielectric DBRs, and m is the number of pairs. To achieve *R* > 99%, the minimum m can be estimated by Equation (3). The corresponding Δλ_stop_, total thickness, and representative reflectivity spectrum are plotted in Figure 3. Apparently, the epitaxial DBR possessed a lower bandwidth and a higher total thickness, which imposes a stringent criterion for strain management (except for the Al_0.82_In_0.12_N/GaN DBR, which is the lattice-matched condition). As a result, current nitride VCSELs adopted either one (hybrid-type) or none (dual-dielectric type) epitaxial mirrors as reflectors. Each approach also encountered its own difficulties in the fabrication process.

**Figure 4:**
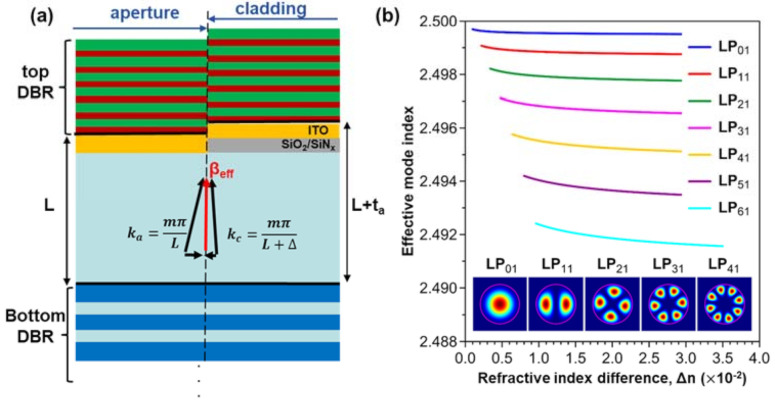
(**a**) Schematic illustration of anti-guiding effect due to the dielectric aperture (**b**) Calculated mode effective indices of LP_mn_ modes of a 6-μm-diameter nitride VCSEL with varying index difference (Δn) between aperture and cladding region 2. The carrier aperture dimension in (**b**) follows the reference [24] (Data from [24]).

Besides, AlGaN in III-nitride VCSELs cannot be easily oxidized to form a carrier aperture as AlGaAs could. The ion implantation process was neither a common process for III-nitride in the beginning. As a result, researchers deposited dielectric (usually SiO_2_) on the bare epitaxial wafer as the carrier aperture. However, the parasitic anti-guiding effect in the lateral direction was not addressed until 2014 [58,59]. Figure 4a schematically illustrated the origin of additional scattering loss due to the dielectric aperture. Assuming the total thickness between two DBR mirrors to be L in the aperture region (including nitride epi and ITO contact), the total thickness in the cladding region is L + Δ, where Δ is the dielectric layer thickness. The k-vectors of standing-wave in the Fabry-Perot cavity gives:

(5) $$k_{a}=\frac{m\pi }{L}$$

(6) $$k_{c}=\frac{m\pi }{L+\Delta }$$

where *m* is the mode number. Since Δ << L, we can reasonably assume that *m* is the same integer in both regions and *k_a_* > *k_c_*. Therefore, while constructing the propagation mode with a common effective mode propagating constant (β_eff_), the oblique angle of the k-vector in the aperture region will be larger than that in the cladding region. As a result, the optical energy will be constantly leaking out of the cavity in the lateral direction during oscillation, which is also known as the anti-guiding effect. The anti-guiding effect is more significant for high-order linear polarized (LP) modes due to a smaller β_eff_, as simulated in Figure 4b. The anti-guiding effect causes a prevailing fundamental mode (LP_01_) from other high-order modes, which might be desired for some applications, but the device performance such as J_th_ and P_max_ will be limited. According to Hashemi et al.’s analysis, the lateral anti-guiding effect contributes ~50% of total modal loss for fundamental LP_01_ mode and ~70% of total modal loss for LP_11_ mode [63]. In other words, the threshold modal gain is increased at least two-fold due to the dielectric aperture.

In this report, the progress of two different types of nitrides VCSELs will be reviewed in session II. Proven strategies in top mirror fabrication to overcome the anti-guiding effects will be discussed in session III.

## 2. Category of III-Nitride VCSELs

**Figure 5:**
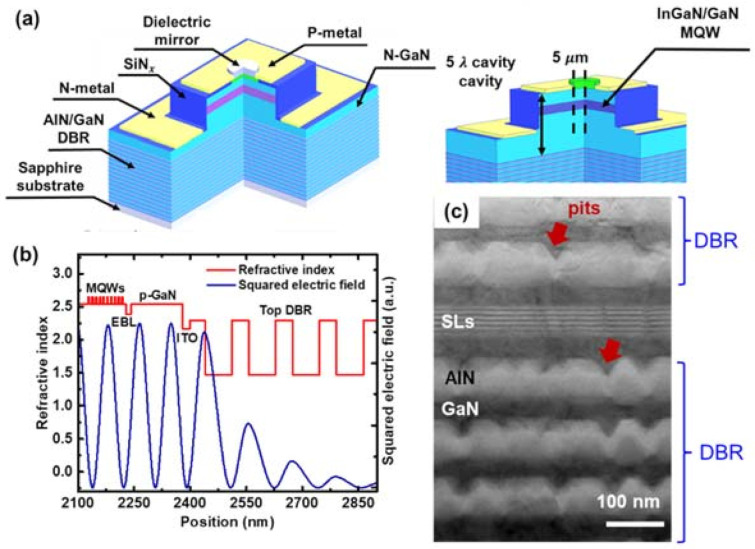
(**a**) Schematic structure and (**b**) optical mode intensity profile of first hybrid-type nitride VCSEL. (**c**) TEM observation of a defective area in AlN/GaN DBR. The epilayer structure for optical simulation of (**b**) followed the reference [12] (Data from [12]).

As aforementioned, III-nitride VCSELs are categorized into 2 types according to the combination of both end mirrors. The hybrid-type VCSEL possesses one mirror by epitaxial growth, and the other by dielectric deposition. The first demonstration is by T.C. Lu et al., and the schematic device structure is shown in Figure 5a,b. The device consisted of 29 pairs of AlN/GaN epitaxial DBR mirrors at n-side, 5λ-long GaN cavity with InGaN multiple-quantum-wells (MQW) at the anti-node, λ/8-thick ITO at the node, and 8 pairs of SiO_2_/Ta_2_O_5_ dielectric mirrors at top. Although short-period AlN/GaN superlattices (SLs) were inserted after every 5 pairs of AlN/GaN DBR to alleviate the strain energy accumulation, strain-induced defects were still observed. Figure 5c showed a transmission electron microscopy (TEM) image of an AlN/GaN DBR. The high density of surface depression (a.k.a. pits) was observed on the top surface of AlN only, which is attributed to the high tensile strain of AlN on the GaN templates. The pits with a central screw dislocation are strong carrier leakage paths. Therefore, the first nitride VCSEL can only be operated under low temperature to retain a high carrier density in MQW.

**Figure 6:**
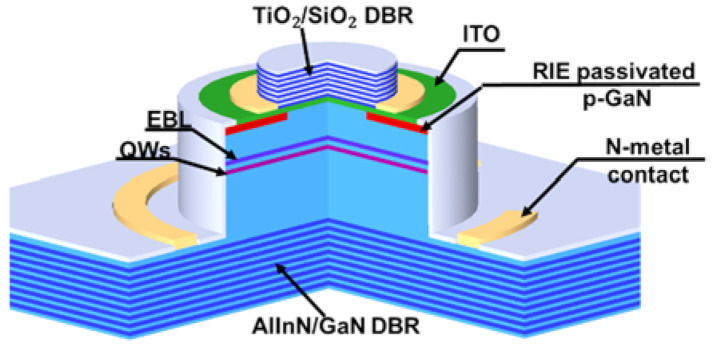
Schematic structure of first hybrid-type VCSEL with AlInN/GaN DBR.

Therefore, the advancement of hybrid-type nitride DBR focused on circumventing the epitaxial strain. The most common approach is to replace the AlN with AlInN lattice-matched to GaN. According to Vegard’s Law, the Al% of lattice-matched AlInN is around 82%. However, the AlInN epitaxial growth is critical because the ideal growth conditions of AlN and InN are way apart, and its strain status is sensitive to the composition variation. Gas-phase reaction, surface pits, and phase separation were all reported for a single AlInN layer growth on GaN [64,65,66,67]. Besides, the optimal growth temperature of AlInN (T_g_ = 850 ~ 900 °C) also differed much from that of GaN (T_g_ = 1050 ~ 1100 °C). Therefore, improper treatment at AlInN/GaN growth interface could cause either In desorption or In segregation at the interface [68,69,70]. High reflectivity lattice-matched AlInN/GaN DBR was demonstrated early in 2003 as in ref. [71], but full VCSELs with lattice-matched AlInN/GaN DBR were not demonstrated until 2012. The schematic structure of the first nitride VCSEL with AlInN/GaN bottom DBR is shown in Figure 6 [21]. The large time gap between DBR and VCSEL demonstration might also be attributed to the significant challenges in the epitaxial growth integration. To date, the single VCSEL with the highest P_max_ (>10 mW) utilized lattice-matched AlInN/GaN DBR. If the challenges in growth can be properly dealt with, nitride VCSEL array with epitaxial DBR is promising for high-power application [42].

Another strategy to fabricate strain-free epitaxial DBRs is by forming nanoporous GaN via electrochemical (EC) etching. Unintentionally doped GaN (UID-GaN) and heavily doped n-GaN (n^+^-GaN) layers were grown alternatively with designed thicknesses, followed by common VCSEL device epi as described in Figure 5. The epi layer is etched to expose the sidewall of UID/n^+^-GaN layers, then the substrate is positively biased in the buffered HF solution for EC etching [72]. Because of the different conductivity between UID-GaN and n^+^-GaN, it’s possible to only porosity the n^+^-GaN layers with deliberately chosen bias voltage and HF concentration. The index contrast between un-etched u-GaN and porous n^+^-GaN can be up to 0.5~0.8, which is in the same level of dielectric DBR’s in Table 2. As a result, the required total thickness is also narrower than the epitaxial DBR’s. Nano-porous III-nitride DBR was firstly demonstrated by J. Han et al. [73]. Optically pumped lasing of nitride VCSELs were demonstrated in 2015, and the electrically pumped lasing is demonstrated by Masabih et al. in 2019 [51,74,75]. The most significant advantage of nano-porous DBR is the ease of growth. Common n-GaN growth conditions in blue LEDs can be directly applied. However, integrating such DBR into real devices could still be challenging since the above device epi must not be EC-etched. The passivation layer must sustain a ~10-volt reverse-bias stress in HF solution. Another challenge for the nano-porous DBR is the low thermal conductivity with air voids, which might inherently limit the P_max_ performance.

**Figure 7:**
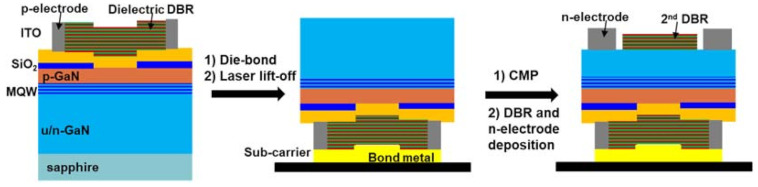
Schematic fabrication process of firstly demonstrated dual-type nitride VCSEL.

Dual-type VCSELs possess deposited dielectric DBRs from both sides. The major challenge lies in the exposure of the bottom surface with a thickness precision. The first dual-type III-nitride DBR was demonstrated by Nichia Corp., and the schematic process route is illustrated in Figure 7 [11]. VCSEL epi was grown on sapphire or GaN and transferred to a sub-carrier via die-bonding and laser lift-off (LLO) or chemical-mechanical polishing (CMP) processes. To reduce the epi thickness to the target cavity length, the transferred epi was thinned via CMP. The process was critical for multiple reasons: (1) conventional LLO process is not applicable to GaN homoepitaxy. The GaN heteroepitaxy on sapphire has its limit in crystal quality. (2) Lack of feedback design to stop the CMP process. As the tolerance of cavity length error becomes narrow for short cavity lasers, residual thickness control will be extremely challenging. (3) The CMP still damages the epilayer near the surface. Restoring the electric contact characteristic requires other engineering efforts.

**Figure 8:**
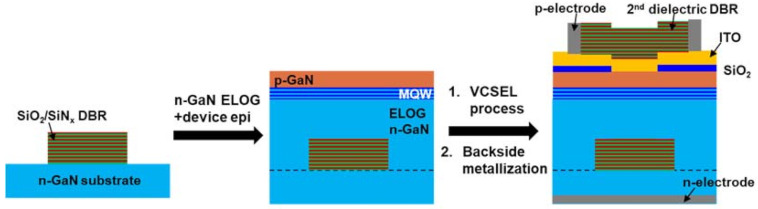
Schematic process route of dual-type VCSEL with ELOG bottom mirror.

**Figure 9:**
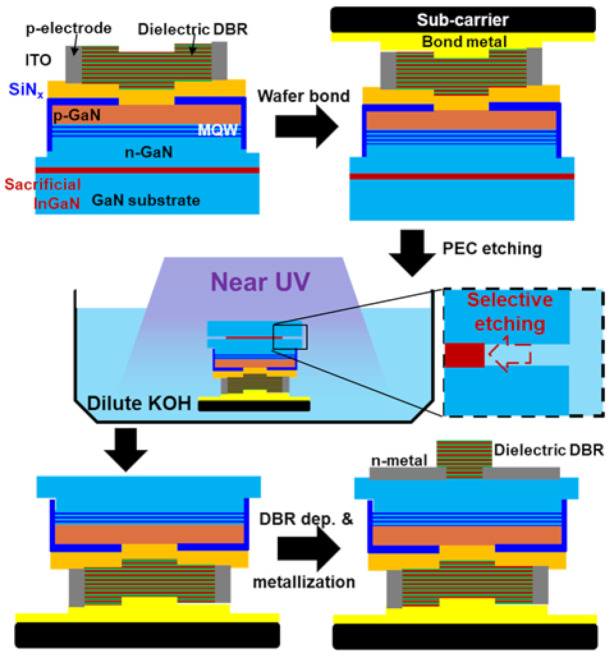
Schematic process of dual-type VCSEL via PEC-etching.

In 2015, Izumi et al. demonstrated dual-type DBR by epitaxial lateral overgrowth (ELOG) on pre-deposited dielectric DBRs as depicted in Figure 8 [35]. A 14.5-pair SiO_2_/SiN_x_ DBR was deposited and patterned on the high-quality n-GaN substrate, and the n-contact was directly formed on the substrate backside. The rest of the device design and fabrication process on the top side is similar. Although the film coalescence and thickness control on the ELOG n-GaN could still be challenging, the crystal quality and the backside contact characteristics were significantly improved. Figure 9 demonstrated another potential route for defining the cavity length with photoelectricalchemical (PEC) etching [76,77,78]. Prior to the device epitaxy, an InGaN sacrificial layer with a near-UV bandgap (λ = 385 nm ~ 405 nm) was grown on a bulk GaN substrate. After a similar VCSEL process was done from the top side, another deep-etch was performed to expose the sidewall of the sacrificial layer. The wafer was then bonded to another sub-carrier. Under near-UV illumination, only the sacrificial layer dissolves in KOH solution. After the sacrificial layer was completely etched, devices were transferred to the sub-carrier with a well-defined cavity length and were ready for the processes at the backside. Electrically pumped PEC-transferred III-nitride VCSEL was first demonstrated by C. Holder et al. and was continuously improved in the same affiliation [44,45,46,47,48,49,50]. In comparison with the ELOG-DBR stack with a micrometer in height and ~10 micrometer in width, cavity length control with a planar sacrificial layer is much less critical. However, the PEC etching rate could be still limited by the carrier and materials transport after the undercut goes deep; passivation of the device epi is also essential for the yield.

In 2018, T. Hamaguchi et al. demonstrated a curved DBR mirror at the backside substrate to form a stable cavity with a long cavity length (>50 μm). [37,38] The lens was originally formed by re-flowing the resin plates on the substrate backside, and the shape was transferred to GaN via dry etching. The curved mirror focuses the propagating Gaussian beam back to the active region. Therefore, the diffraction loss in a long cavity could be minimized. Although the presented P_max_ still has room for improvement, the latest J_th_ was reported as low as 4 kA/cm^2^. Besides, a long cavity has a strong potential for mass production. For example, the much narrower mode spacing makes the cavity length tolerance irrelevant; a thick base is also beneficial for thermal management and is more robust for packaging.

## 3. Novel Carrier Aperture Designs

**Figure 10:**
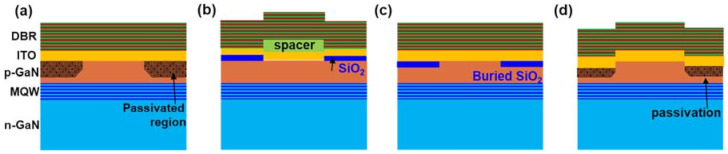
Schematic aperture designs with (**a**) ion-implantation or plasma passivation (**b**) additional spacer (**c**) buried SiO_2_ and (**d**) plasma etched and passivated cladding region for lateral optical confinement.

**Figure 11:**
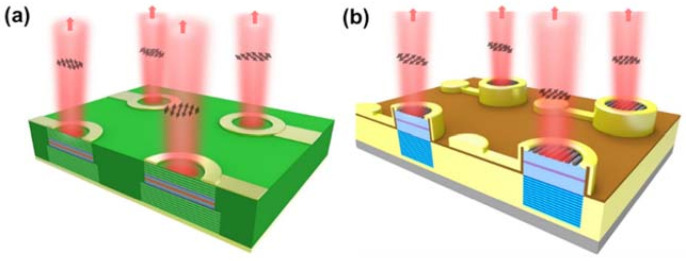
(**a**) Lasing with random polarization in conventional VCSEL arrays (**b**) lasing with pre-designed polarization by high-contrast grating (HCG) mirror.

To overcome the anti-guiding effects due to the deposited dielectric aperture, the optical path difference between the central region and cladding region needs to be compensated. An intuitive approach is to get rid of the dielectric mirror by passivating the cladding region by ion-implantation or plasma treatment. The schematic aperture design was shown in Figure 10a [37,38,43,45,49,50,51]. As a result, the vertical optical path between mirrors in both regions is identical. One could also enhance the optical path in the central region with an additional spacer as depicted in Figure 11b [79], or etch some p-GaN off in the cladding region as shown in Figure 10c,d [39,40,41,42,43]. If the optical path in the central region becomes longer than that of the cladding region instead, the lateral optical confinement alters from anti-guiding to guiding. Figure 10c,d both etched some p-GaN off, but (c) still adopted a dielectric layer to block the current (buried aperture) while (d) directly exploited the plasma-damaged surface as the carrier aperture. From the perspective mirror curvature, the original dielectric aperture yielded a concave-like mirror, aperture in Figure 10a yielded a planar mirror, and designs in Figure 10b–d yielded convex-like top mirrors.

With novel apertures in Figure 10c,d, the maximum output power is greatly enhanced. Single VCSEL demonstrated P_max_ > 5 mW performance and 16 × 16 VCSEL array reached P_max_ > 1 W [42] The maximum output power of III-nitride VCSELs is no longer less than the arsenide and phosphide-based VCSELs. The side effect of novel aperture design is the loss of discrimination to higher-order modes [24]. As shown in Figure 4b as the effective refractive index difference (Δn) between the central region and cladding region becomes positive, optical modes with higher azimuthal order are allowed. Fortunately, multi-mode lasing is not a critical issue for most blue VCSEL applications.

## 4. Polarization Control of III-Nitride VCSEL

Forming a VCSEL array is the most cost-effective way to push the P_max_ up to another order. Unlike the TE-mode lasing is strongly preferred in EELs, the polarization in the VCSEL array on the basal plane is random as shown in Figure 11a. If the applications require lasing in specific polarization, for example, holographic projection and polarization-multiplexing in visible communication, the final device output power will be reduced in half after including an external linear polarizer. Therefore, polarization control is also an interesting topic for VCSEL applications. Although manipulating the polarization to an arbitrary angle is difficult, it is possible to restrict polarization in the VCSEL array in the same direction as depicted in Figure 11b.

The randomness of polarization originates from the circularly invariant gain and loss in the VCSEL structure. To discriminate one polarization mode from the other, we must differentiate their optical gain or losses. For example, growing the VCSEL structure in nonpolar and semipolar planes, or replacing the DBR with high contact grating (HCG) were proven effective in reducing the circular symmetry without changing the shape of the aperture.

**Figure 12:**
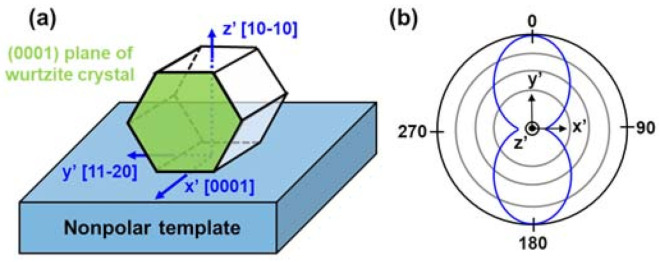
(**a**) Illustration of x’,y’ and z’ orientation in non-polar epitaxy system as an example. (**b**) Schematic illustration of polarization of non-polar VCSELs when I > I_th_.

Growing devices on nonpolar planes or semipolar planes mitigate QCSE. Saturated optical gain is also enhanced due to the improved wavefunction overlap and transition matrix elements. [80,81] However, the origin of polarization discrimination comes from the anisotropic strain along the a-axis and c-axis. Because the c/a ratio within AlN, GaN, and InN are all different, AlGaN (or InGaN) on the GaN template will experience a different amount of strain along with the a-axis and c-axis projection. The strain of AlN and InN on GaN within the basal plane (ε_x_) and along the c-axis (ε_z_) were also summarized in Table 1. Figure 12a depicted the nomenclature of orientations in the non-polar and semipolar epitaxy system. z’ is the growth normal direction, y’ is parallel to the intersection of the growth plane and (0001) basal plane, and x’ is parallel to the c-axis projection on the growth plane. For example, the x’, y’, and z’ direction of the nonpolar (10–10) plane is [0001], [11–20], and [10–10], respectively. For the semipolar (20–2–1) plane, the x’, y’, and z’ direction is [10–14], [1–210], and [20–2–1], respectively. Theoretical band structure calculation predicted the top four valence sub-bands are more y’-polarized, only the bottom two sub-bands are more x’-polarized [82]. Non-polar and semipolar LED studies also verified that the spontaneous emission is y’-polarized, and the degree of polarization (DOP) is enhanced with a higher indium content in the active region [83,84,85]. Therefore, the optical gain of y’-polarized light will also be much higher than the x’-polarized light due to the prevailing transition matrix element and population inversion factor, f_c_–f_v_. Blue and violet VCSEL on (10–10) and (20–2–1) planes demonstrated a ~100% DOP after lasing, and their polarization was all along the [1–210] direction [45,46,47,48,49]. Figure 12b schematically illustrated the polarization intensity profile of nonpolar and semipolar III-nitride VCSELs.

High contrast grating (HCG) mirror was firstly demonstrated in arsenide-based VCSELs in the form of the suspending GaAs bars [86]. The reflectivity and the stop-band will be higher and broader if the refractive index difference between grating materials and cladding materials (mostly air) is high. If the periodicity of the grating (Λ) is in the sub-wavelength region, the parallel bars could be regarded as optically coupled slab waveguides with a common mode propagation constant β. Under a specific combination of the grating thickness (h) and bar width (w), the boundary conditions might yield a pure imaginary β, or the imaginary part of β is much higher than its real part [87,88,89]. Under such a combination of grating parameters, the incident wave could not (or barely could) propagate in the HCG region, resulting in a high reflectance of the incident wave.

**Figure 13:**
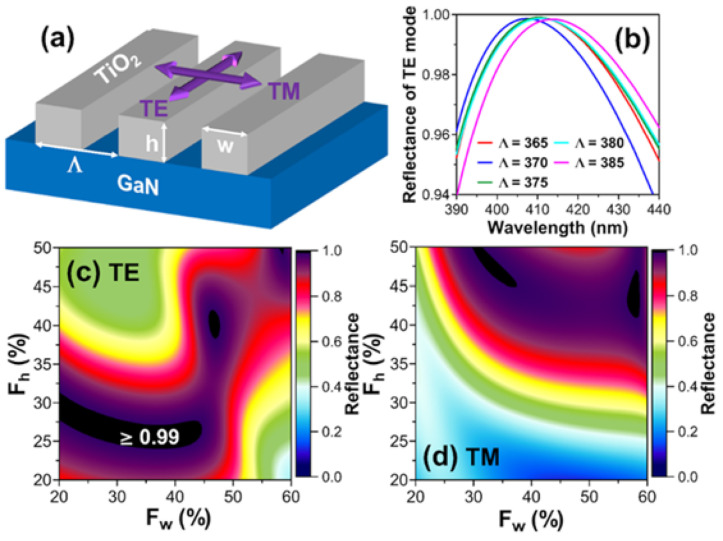
(**a**) Schematic illustration of TE-mode and TM-mode polarization in TiO_2_ HCG on GaN (**b**) Reflectance spectrum of selected HCG structures. Reflectance mapping in the F_w_/F_h_ parameter space with (**c**) TE-mode and (**d**) TM-mode polarization.

Since suspending GaN bars in the sub-wavelength scale might be quite difficult to fabricate, a patterned high index dielectric could be a practical alternative. Chang et.al demonstrated optically pumped and electrically pumped III-nitride VCSEL with a sub-wavelength TiO_2_ grating replacing conventional dielectric DBRs [90,91]. The lasing modes are either parallel (TE-mode) or perpendicular (TM-mode) to the TiO_2_ bars as depicted in Figure 13a. Figure 13b–d are calculated reflection spectrum and reflectivity mapping with different grating parameters. F_w_ and F_h_ are normalized grating parameters defined by:

(7) $$F_{w}=\frac{w}{\Lambda }$$

(8) $$F_{h}=\frac{h}{\Lambda }$$

The selected grating structures with Λ from 365 nm to 385 nm in Figure 13b were summarized in Table 4. With a proper design, R > 99% with ~20 nm bandwidth could be achieved. Figure 13c,d plotted the reflectivity mapping with varying F_w_ and F_h_ under Λ = 375 nm and λ = 410 nm. Because the boundary conditions between TE-mode and TM-mode are different, the parameter design space for high reflectance also differs. If the combination of (F_w_, F_h_) only gives a high R to one polarization mode, the other polarization will be discriminated for lasing. For example, if (F_w_, F_h_) in Figure 13c,d is (30%, 28%), R for TE-mode exceeds 99% while R for TM-mode is less than 40%. As a result, TE-mode lasing prevails.

## 5. Outlook of III-Nitride VCSEL Research

After a decade of solid development, blue III-nitride VCSELs have achieved remarkable progress in fabrication technologies and device performances. New applications with III-nitride VCSELs such as VR/AR displays and micro-projectors are also on the way to market. In near future, the frontier of III-nitride VCSEL research will keep advancing in various dimension; for example, higher power density, broader applicable spectral region, or much lower threshold pumping level.

To further enhance the output power density, one might consider stacking multiple VCSEL device epilayers with tunnel junctions. Tunnel junction has been applied to single III-nitride EELs to circumvent the relatively poor current spreading and contact resistances of p-GaN [92]. A similar concept was also demonstrated with single III-nitride VCSEL [93]. Combining the vertical device epi stack and lateral array structure, the absolute output power from a monolithic VCSEL array chip has the potential to exceed the output power of EELs.

III-nitride materials also have the potential for fabricating VCSELs in a spectral region other than blue. The bottleneck for III-nitride VCSELs working in longer wavelengths is in growing high In-content QWs. The strong compressive strain in QW might trigger catastrophic materials degradation such as phase separation or macroscopic voids [94,95,96,97]. Since nitride-based aquamarine to red EELs and green VCSELs have been demonstrated, there shall be no real technology bottleneck in the current stage [98,99,100]. However, since the index contrast between AlInN and GaN is weaker and the Bragg’s wavelength is longer, the epitaxial DBR growth will be more critical as well. Fulfillment of R-G-B VCSEL triggers new design possibilities in VR/AR headset/glasses or compact-sized holographic projectors. In other directions, III-nitride is also the most promising candidate in fabricating ultra-violet (UV) VCSELs. The challenge in growth becomes the strain and morphology management with Al-rich AlGaN. Similarly, since electrically pumped nitride EELs have been demonstrated from 270 nm to 405 nm, [101,102] growth of high-quality AlGaN active region shall not be the limiting bottleneck either [103,104,105,106]. However, the DBR technology for the UV spectral region needs further development. Many high-index dielectrics such as TiO_2_ are strongly absorbing under UV light, and GaN cannot be used in UVB/UVC epitaxial DBR due to the band edge absorption. Therefore, the cavity design and fabrication of UV VCSELs will be even more critical than that of long-wavelength VCSELs. UV VCSELs can be used as an alternative component in an atomic clock, UV resin-curing, gas sensing, and disinfections [107,108,109].

To further reduce the threshold pumping level, the lateral optical confinement requires a more progressive design; for example, optically coupled monolithic nano-rod arrays [110,111]. Because crystallographic facets naturally form during the regrowth of III-nitride nanowires, DBRs are not necessary for a high-Q cavity. Optically pumped and electrically pumped lasing of coupled III-nitride nano-rod array have been demonstrated with a low pumping density [112,113,114]. Since the photons were strongly confined in the lateral direction, photons after lasing still emit along the surface normal. Another approach is to fabricate exciton-polariton lasers for the ultra-low threshold pumping density. Although the structure of the exciton-polariton laser resembles the VCSEL structure, the fundamental physical mechanism is totally different. Exciton-polariton lasing originates from the strong coupling between cavity photon and bounded exciton. The bosonic nature allows excitons in the cavity to condensate in the same energy level, and the phase is equalized by the standing photon waves [115]. Therefore, its theoretical pumping level could be ordered lower than that of photonic lasers because population inversion is not required. Electrically pumped polariton laser has been demonstrated in GaAs-based materials, [116,117] exciton-polariton coupling was also observed under optical pumping in the nitride system [118,119,120]. Because the binding energy of exciton in GaAs is low (~5 meV), polariton-exciton lasing under room temperature is highly unlikely. AlGaN materials possess a high exciton binding energy from 25 meV to 60 meV, which is very promising for an exciton-polariton laser operating above room temperature [121]. The realization of room-temperature exciton-polariton laser provides a platform for studying the multiple-body effects in a bosonic system, which might be essential for the development of quantum information technologies.

In conclusion, III-nitride VCSELs have encountered many challenges and opportunities due to their unique materials properties. High-power blue VCSELs have been demonstrated after breakthroughs in mirror and aperture fabrication technology. The authors believe the scope and functionality of III-nitride VCSELs and their derivatives will keep expanding in all dimensions in the upcoming future.

## Figures and Tables

**Table 1:** The lattice constant, spontaneous polarization, piezoelectric tensor elements, elastic constant, electron effective masses, and of wurtzite nitride materials. Parameters collected from references [13,14,15,16,17].

	AlN	GaN	InN
a_0_ (Å)	3.112	3.186	3.548
c_0_ (Å)	4.982	5.186	5.703
ε_x_ to GaN (%)	2.47	--	−10.20
ε_z_ to GaN (%)	4.07	--	−9.07
P_sp_ (C/m^2^)	−0.081	−0.029	−0.032
e_31_ (C/m^2^)	1.46	0.73	0.97
e_33_ (C/m^2^)	−0.60	−0.49	−0.57
C_13_ (GPa)	108	103	92
C_33_ (GPa)	373	405	224
m_e_/m_0_	0.4	0.2	0.11

**Table 2:** Structure and device summary of blue VCSEL benchmarks of Figure 2. In the “Reflectors” columns, “Hybrid” implied one reflector is grown epitaxially and the other is deposited dielectrics. Dual dielectric (epitaxial) means both reflectors were deposited dielectrics (epitaxial mirror). TJ in the carrier aperture column stands for tunnel junction. If the same affiliation reported different device results with the same device configuration, only the latest published result will be recorded in the table. * Representative near-IR device VCSEL results were put at the bottom of the table. For a more compact table, the spectral region is recorded in the affiliation column.

Affiliation	Reflectors	Cavity Length	Carrier Aperture	J_th_ (kA/cm^2^)	P_max_ (mW)	Operating Condition	Ref.
NCTU, Hsinchu, Taiwan	Hybrid (AlN/GaN)	5 λ	Flat SiO_2_	10.6	0.9	CW/RT	[12,27,28]
	Dual dielectric	2.5 λ		8.9	0.02	Pulse/350 K	[29,30]
	Dielectric + HCG	5 λ		11.2	0.007	Pulse/RT	[31]
Nichia Corp., Tokushima, Japan	Dual dielectric	7 λ	Flat SiO_2_	1.9	0.7	CW/RT	[11,32,33]
Sony Corp., Kanagawa, Japan	Dual dielectric	10 λ	Flat SiO_2_	15.9	0.9	CW/RT	[35,36]
		~150 μm	Implanted B	4.2	0.3		[37,38]
Panasonic Corp. Kyoto, Japan	Dual dielectric	30 λ	Flat SiO_2_	0.5	0.003	CW/RT	[34]
EPFL, Lausanne, Swiss	Hybrid (AlInN/GaN)	7 λ	RIE passivation	139.3	0.35	CW/RT	[21]
Meijo Univ. & Nagoya Univ., Nagoya, Japan	Hybrid (AlInN/GaN)	1.5 λ ~ 4.5 λ	Flat SiO_2_	4.0	0.88	CW/RT	[22,23,24,25,26]
Stanley Electric Corp. Tokyo, Japan	Hybrid (AlInN/GaN)	4.5 λ~10 λ	Buried SiO_2_	11.6	15.7	CW/RT	[39,40,41,42]
		10 λ	RIE passivation	21.3	23.7	CW/RT	[43]
Univ. of California, Santa Barbara, CA, USA	Dual dielectric	7.5 λ	Flat SiN_x_	90.9	0.019	Pulse/RT	[44]
		7 λ	Implanted Al	17.7	0.012		[45]
		7 λ	Ta_2_O_5_ with TJ	3.5	0.55		[46]
		7 λ	PEC air-gap	22.0	0.18		[47,48]
		6.5 λ ~ 23 λ	Implanted Al	9.7	0.319		[49,50]
Univ. of New Mexico Albuquerque, NM, USA	Hybrid (GaN/ nano-porous GaN)	8 λ	Implanted Al with TJ	20.0	1.5	Pulse/RT	[51]
Xiamen Univ., Xiamen, China	Dual dielectrics	NA	Flat SiO_2_	1.2	0.5	CW/RT	[52]
		2.5 λ	Buried SiO_2_	9.1	0.2		[53]
* 850 nm	**Dual epitaxial**	**NA**	**Al_x_O_y_ oxide**	**0.9**	**9**	**CW/RT**	**[54]**
**940 nm**		**NA**		**1.7**	**7.7**		**[55]**
**980 nm**		**0.5 λ**		**0.8**	**11**		**[56]**
**1310 nm**		**2.5 λ**	**TJ**	**3.1**	**3.6**		**[57]**
**1550 nm**	**Dual dielectric**	**2.5 λ**		**3.5**	**3.0**		**[58]**

**Table 3:** The refractive constants of common materials used in nitride VCSELs. Parameters collected from references [59,60,61,62].

Dielectric DBR	SiO_2_	SiN_x_	Ta_2_O_5_	ZrO_2_	TiO_2_	Nb_2_O_5_
n at 420 nm	1.468	1.956	2.228	2.211	2.294	2.501
**Epitaxial DBR**	**GaN**	**Al_0.6_Ga_0.4_N**	**Al_0.82_In_0.18_N**		**AlN**	
n at 420 nm	2.545	2.255	2.135		2.096	

**Table 4:** The grating parameters of Figure 13b.

Λ (nm)	F_w_ (%)	F_h_ (%)
365	18.0	31.5
370	21.0	29.5
375	29.0	26.5
380	31.5	25.5
385	34.5	25.0

## Data Availability

Not applicable.
